# Upregulation of CD38 expression on multiple myeloma cells by novel HDAC6 inhibitors is a class effect and augments the efficacy of daratumumab

**DOI:** 10.1038/s41375-020-0840-y

**Published:** 2020-04-29

**Authors:** Estefanía García-Guerrero, Ralph Götz, Sören Doose, Markus Sauer, Alfonso Rodríguez-Gil, Thomas Nerreter, K. Martin Kortüm, José A. Pérez-Simón, Hermann Einsele, Michael Hudecek, Sophia Danhof

**Affiliations:** 1 Instituto de Biomedicina de Sevilla (IBIS), Hospital Universitario Virgen del Rocío/CSIC/Universidad de Sevilla, Sevilla, Spain; 2 Department of Biotechnology and Biophysics, Biocenter, and RVZ for Integrative and Translational BioImaging, Julius-Maximilians-Universität Würzburg, Würzburg, Germany; 3 Medizinische Klinik und Poliklinik II, Universitätsklinikum Würzburg, Würzburg, Germany

**Keywords:** Combination drug therapy, Myeloma, Immunotherapy

## Abstract

Multiple myeloma (MM) is incurable, so there is a significant unmet need for effective therapy for patients with relapsed or refractory disease. This situation has not changed despite the recent approval of the anti-CD38 antibody daratumumab, one of the most potent agents in MM treatment. The efficiency of daratumumab might be improved by combining it with synergistic anti-MM agents. We therefore investigated the potential of the histone deacetylase (HDAC) inhibitor ricolinostat to up-regulate CD38 on MM cells, thereby enhancing the performance of CD38-specific therapies. Using quantitative reverse transcription polymerase chain reaction and flow cytometry, we observed that ricolinostat significantly increases CD38 RNA levels and CD38 surface expression on MM cells. Super-resolution microscopy imaging of MM cells by *direct* stochastic optical reconstruction microscopy confirmed this rise with molecular resolution and revealed homogeneous distribution of CD38 molecules on the cell membrane. Particularly important is that combining ricolinostat with daratumumab induced enhanced lysis of MM cells. We also evaluated next-generation HDAC6 inhibitors (ACY-241, WT-161) and observed similar increase of CD38 levels suggesting that the upregulation of CD38 expression on MM cells by HDAC6 inhibitors is a class effect. This proof-of-concept illustrates the potential benefit of combining HDAC6 inhibitors and CD38-directed immunotherapy for MM treatment.

## Introduction

Multiple myeloma (MM) is a plasma cell neoplastic disease with a median overall survival of 4.4–7.1 years that often runs an aggressive and incurable course [1]. The treatment of MM has improved remarkably over the last 15 years because of the development of novel agents such as proteasome inhibitors (PIs) and immunomodulatory drugs (IMiDs) [1–3]. Nevertheless, MM remains an incurable disease as patients continue to relapse upon treatment. It is therefore necessary to develop more efficient MM therapies, especially for people who have relapsed after treatment with PIs and IMiDs [4, 5].

For these patients, the anti-CD38 monoclonal antibody (mAb) daratumumab was first approved in 2015 as a single agent therapy [6, 7]. CD38 is highly and ubiquitously expressed on MM cells and at low levels on normal lymphoid and myeloid cells [8]. CD38 is a transmembrane glycoprotein with ectoenzymatic activity in the catabolism of extracellular nucleotides [9]. Other functions include receptor-mediated adhesion by interacting with CD31 or hyaluronic acid, regulation of migration, and signaling events [10]. In patients with relapsed/refractory (R/R) MM, daratumumab monotherapy produces only a low rate of partial and complete remission (29.2%), and a limited median duration of response (7.4 months) [7, 11]. Therefore, daratumumab is increasingly being used in combination with bortezomib/dexamethasone (dex) or lenalidomide/dex at first relapse. Such combined treatment induces a complete response (CR) in a notable percentage of patients (19.2% with dex and 43.1% with lenalidomide/dex) [12, 13]. More recently, daratumumab has also been approved as a third-line treatment in combination with pomalidomide/dex (CR rate 17%) and as a first-line treatment in combination with bortezomib/melphalan/prednisolone (CR rate 43%) [14]. However, although combination therapies using daratumumab belong to the most potent regimens currently available, there are still relapsing or refractory patients [12–14].

Clinical observations with daratumumab are that the expression of CD38 on pretreated MM cells correlates with efficacy and that patients with higher CD38 expression are more likely to respond [15]. Moreover, during daratumumab therapy, CD38 expression levels on MM cells decline thus favoring immune escape and disease progression [15]. These observations suggest that an increase in the density of CD38 molecules on MM cells is likely to improve response rates and response durability of daratumumab treatment, and prevent immune escape. Therefore, a great amount of research is being invested in defining agents that increase CD38 expression on MM cells and work synergistically with daratumumab. As part of this research, we have recently shown that panobinostat induces CD38 upregulation and augments the antibody-dependent cellular cytotoxicity (ADCC) of daratumumab [16]. Panobinostat is the only histone deacetylase (HDAC) inhibitor approved for myeloma treatment and non-selectively inhibits all HDAC isoforms. However, as panobinostat is associated with relevant toxicity, its clinical use is currently limited.

In search of HDAC inhibitors with a more beneficial side effect profile than panobinostat, novel agents such as ricolinostat (ACY-1215) have been investigated. Ricolinostat is an orally bioavailable, specific inhibitor of histone deacetylase 6 (HDAC6) with potential antineoplastic activity. Its efficacy was evaluated in a phase 1/2 trial as monotherapy and in combination with bortezomib and dexamethasone in heavily pretreated patients with relapsed or R/R MM [17]. Single agent ricolinostat therapy had a more favorable safety profile than other HDAC inhibitors [18] and the overall response rate of the combination regimen with daily ricolinostat at dose levels ≥160 mg was 37% (14% among bortezomib-refractory patients).

An accurate quantification of the antigen density on cell surfaces can be challenging. However, we have shown that it is possible to use a single-molecule sensitive super-resolution microscopy method termed *direct* stochastic optical reconstruction microscopy (*d*STORM) to precisely quantify even very low amounts of surface antigens in the plasma membrane [19, 20]. *d*STORM uses conventional fluorescently labeled antibodies in combination with a switching buffer to bypass the diffraction limit of standard fluorescence microscopy [19, 21]. *d*STORM achieves a spatial resolution of ~20 nm by photoswitching of organic fluorophores between a bright and a nonfluorescent dark state.

We therefore applied *d*STORM and other techniques to investigate the effect of the HDAC6 inhibitor ricolinostat and the novel HDAC6 inhibitors ACY-241 and WT-161 on CD38 expression levels on myeloma cells and to determine whether upregulation of CD38 is a class effect that works synergistically with daratumumab.

## Material and methods

### Human subjects

Peripheral blood and bone marrow aspirates were obtained from healthy donors and MM patients after we obtained their written informed consent. Patient characteristics are depicted in Supplementary Table 1. The research protocols were approved by the Institutional Review Boards of the University Hospital of Würzburg (UKW) and University Hospital Virgen del Rocío (HUVR). All procedures conformed to the Helsinki Declaration.

### Isolation of primary myeloma cells, regulatory T cells, and T cells

We isolated peripheral blood mononuclear cells (PBMCs) and bone marrow mononuclear cells by density gradient centrifugation using Ficoll–Paque (Amersham Biosciences, Uppsala, Sweden). We isolated primary MM cells from bone marrow aspirates using CD138 immunomagnetic beads (Miltenyi Biotec, Bergisch-Gladbach, Germany). Regulatory T cells were isolated from PBMCs using a two-step immunomagnetic procedure (Miltenyi). T cells were also isolated from PBMCs using negative selection with immunomagnetic beads (Miltenyi). CD8+ and CD4+ T cells were activated by anti-CD3/CD28 bead stimulation (Thermo, Waltham, MA).

### Immunophenotyping

The expression of CD38, BCMA, SLAMF7, CD55, and CD59 on MM cells was analyzed by flow cytometry using specifically conjugated mAbs and matched isotype controls. We stained with 7-aminoactinomycin D (7-AAD) to discriminate between living and dead cells. Flow cytometry analyses were performed on a FACSCanto II (Becton Dickinson, Heidelberg, Germany) and data was analyzed using FlowJo software (Treestar, OR, USA).

### Treatment with HDAC inhibitors and all-trans retinoic acid (ATRA)

We cultured the MM cells in RPMI-1640 (Gibco, Darmstadt, Germany) supplemented with 10% fetal bovine serum at 1 × 10e5 cells/well in 96-well flat-bottom plates (Costar, Washington, DC). We reconstituted ricolinostat, ACY-241 and WT-161 in dimethylsulfoxide and added them to the medium at final concentrations of 1, 5, and 10 µM. We likewise reconstituted ATRA and Panobinostat in dimethylsulfoxide and added these compounds to the medium at final concentrations of 10 nM.

### Antibody-dependent cellular cytotoxicity (ADCC) assay with primary myeloma cells

Ricolinostat-treated (5 µM, 48 h) primary MM cells were co-cultured with autologous PBMCs at an effector-to-target ratio of 3:1 in 96-well plates in the presence of solvent control, IgG1 isotype or daratumumab. After 24 h, the percentage of viable myeloma cells was determined by flow cytometry using 7-AAD to discriminate living from dead cells.

### Statistical analysis

For statistical analyses, we used Prism Software (GraphPad, San Diego, CA). Shapiro–Wilk test was used to test for normality. Unpaired Student’s *t*-tests were used to analyze CD38 expression level. Two-way ANOVA testing was performed to analyze the functional data. Differences with a *p*-value < 0.05 were considered statistically significant.

## Results

### Ricolinostat increases CD38 expression on myeloma cells

**Fig. 1:**
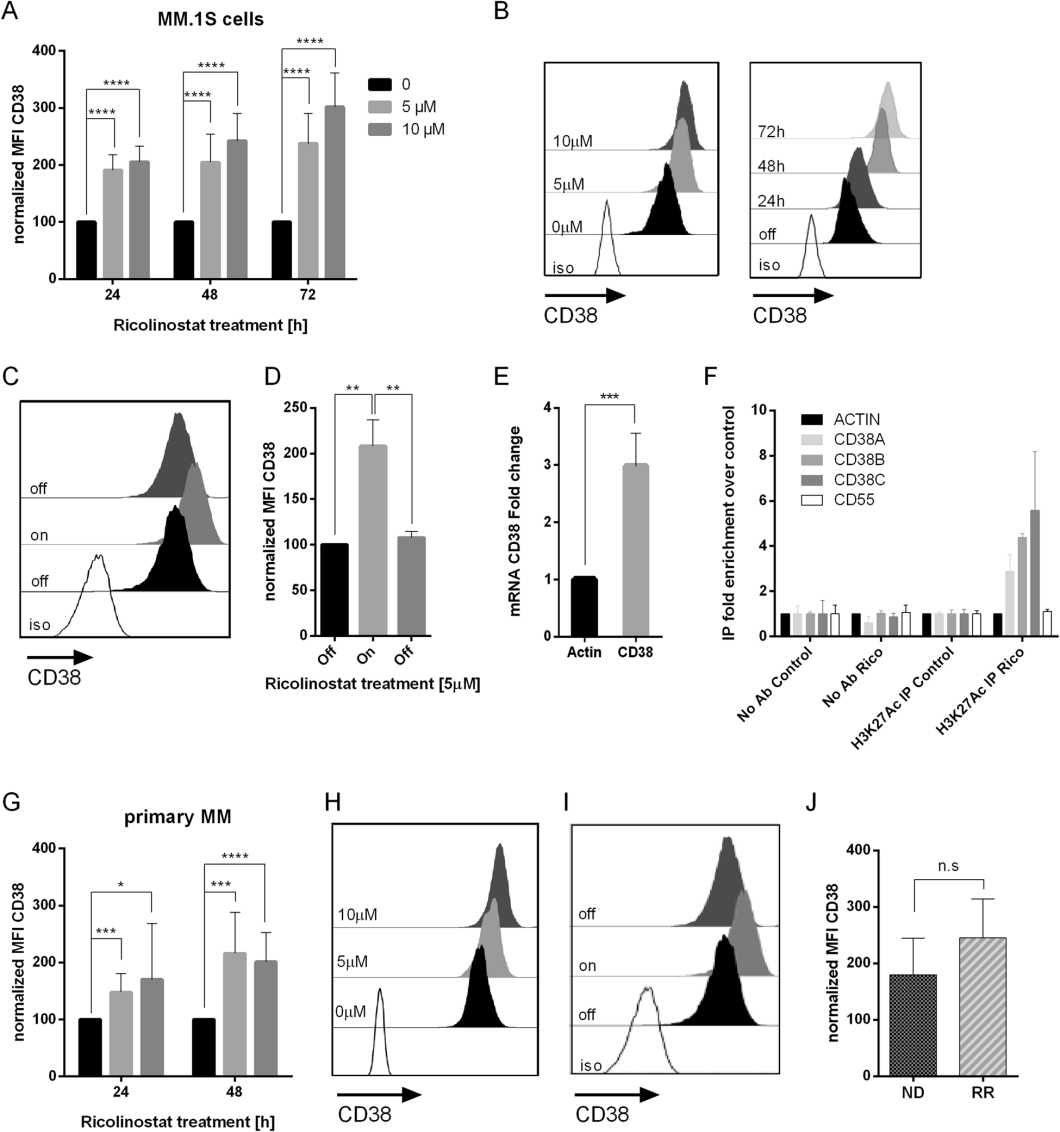
Ricolinostat treatment leads to enhanced CD38 expression on myeloma cells. **a** CD38 expression on MM.1S cells (*n* = 13 experiments) before and after treatment with ricolinostat at the given final concentrations (fc) for the given time intervals. Bar diagram shows CD38 expression as normalized MFI of treated vs. untreated MM.1S cells. **b** The overlay histogram (left) shows flow cytometric analysis of CD38 expression on MM.1S cells cultured in the absence or presence of ricolinostat for 72 h. The overlay histogram (right) shows change in CD38 expression on MM.1S cells during ricolinostat treatment at a fc of 5 µM. **c** The overlay histogram shows CD38 expression on untreated MM.1S cells, 24 h after ricolinostat treatment at a fc of 5 µM, and 72 h after subsequent removal of the drug. **d** Bar diagram shows CD38 expression level as normalized MFI on untreated MM.1S cells (*n* = 3 experiments), 24 h after ricolinostat treatment at a fc of 5 µM, and 72 h after subsequent removal of the drug. **e** CD38 RNA levels on MM.1S cells (*n* = 4 experiments) by quantitative reverse transcription PCR (qRT-PCR) were quantified after incubation with ricolinostat at a fc of 5 µM for 48 h. **f** ChIP of histone 3 lysine 27 acetylation in the CD38 promoter. Chromatin immunoprecipitation was performed on MM.1S cells treated with 5 µM of  ricolinostat for 24 h or untreated control cells, using anti H3K27Ac antibodies, or with no antibodies as control. Immunoprecipitated chromatin was quantified using RT-qPCR with primer pairs for the promoters of Actin (technique control), CD55 (internal control), and three different pairs for CD38. CD55 was used as an internal control because its expression level is not affected by ricolinostat treatment. The immunoprecipitation was calculated normalizing to the input of each sample, the Actin promoter signal and the average of the control samples. Mean with SD of two biological replicates is shown. **g** Bar diagram shows CD38 expression on primary myeloma cells (*n* = 9 patients) before and after ricolinostat treatment. **h** The overlay histogram shows flow cytometric analysis of CD38 expression on primary MM cells cultured in the absence or presence of ricolinostat for 72 h. **i** The overlay histogram shows CD38 expression on untreated primary MM cells, 72 h after ricolinostat treatment at a fc of 5 µM, and 24 h after subsequent removal of the drug. **j** Bar diagram shows CD38 expression of newly diagnosed (ND, *n* = 4 patients) and relapsed/refractory (R/R, *n* = 5 patients) MM patients after 48 h treatment with ricolinostat at a fc of 5 µM. Shaded histograms show staining with anti-CD38 mAb, white histograms show staining with isotype control antibody. 7-AAD was used to exclude dead cells from analysis. Depicted are mean values with SD. *p*-Values between indicated groups were calculated using Student’s *t*-test. n.s. = not significant, *p < 0.05, **p < 0.005, ***p < 0.001, ****p < 0.0001.

We treated the MM cell line MM.1S with the first-in-class HDAC6 inhibitor ricolinostat and analyzed the expression of CD38 on residual live cells. In flow cytometry, there was a 2.5-fold (5 µM) and 3.1-fold (10 µM) increase in CD38 expression at 72 h of treatment by mean fluorescence intensity (MFI) (*p* < 0.0001, *n* = 13) (Fig. 1a, b). The increase in CD38 expression was detectable within 24 h and increased further the longer exposure to the drug lasted (Fig. 1b). It is of note that the withdrawal of ricolinostat resulted in a significant decline of CD38 expression on MM cells to baseline levels within 72 h (Fig. 1c, d). Similar trends were observed on the MM cell line OPM-2 (Supplementary Fig. 1). To further investigate the correlation between CD38 surface density and CD38 gene transcription, we developed a quantitative reverse transcription PCR assay (qRT-PCR) to measure CD38 RNA levels inside the MM.1S cells. After treatment with ricolinostat, CD38 mRNA levels were higher indicating that the increase of surface CD38 was associated with enhanced gene expression (*p* < 0.001, *n* = 4) (Fig. 1e). This result prompted us to perform a chromatin immunoprecipitation (ChIP) assay to elucidate the mechanism by which CD38 is upregulated under ricolinostat treatment. We observed an increased acetylation of histone 3 lysine 27 in the CD38 promoter (Fig. 1f), suggesting that the inhibition of HDAC6 by ricolinostat prevents the deacetylation of the CD38 promoter.

We further confirmed the effect of ricolinostat on primary MM cells. We treated MM cells from nine patients (Supplementary Table 1) with ricolinostat at final concentrations of 5 and 10 µM, respectively, and produced a uniform increase in CD38 expression in each case by flow cytometry (Fig. 1g, h). The upregulation of CD38 was detectable within 24 h and peaked after 48 h of exposure to ricolinostat. At 48 h, the MFI for CD38 expression was more than 2-fold higher in ricolinostat-treated than in untreated primary MM cells (*p* < 0.0001, *n* = 9 patients) (Fig. 1g). Withdrawal of the drug led to a reduction in CD38 expression to baseline levels (Fig. 1i). The increase in CD38 was similar in patients with newly diagnosed (4/9) and (daratumumab-naive) R/R MM previously treated with IMiDs and PIs (5/9) (Fig. 1j).

To determine a CD38-specific effect of ricolinostat, we analyzed the expression of B-cell maturation antigen (BCMA) [22, 23] and SLAM family member 7 (SLAMF7) [24], alternative targets in MM, after ricolinostat treatment. We detected, in contrast to the effects on CD38, stable or reduced expression of BCMA and SLAMF7 on MM cells at all tested doses and time points (Supplementary Fig. 2). Consistent with previous work [17, 25], ricolinostat exerted a direct cytotoxic anti-MM effect in both primary cells and cell lines (Supplementary Fig. 3).

In combination, these results show that treatment with ricolinostat leads to increased CD38 RNA and surface CD38 protein expression levels in MM cells.

### CD38 is homogeneously distributed in the plasma membrane of myeloma cells and the density increases after ricolinostat treatment

**Fig. 2:**
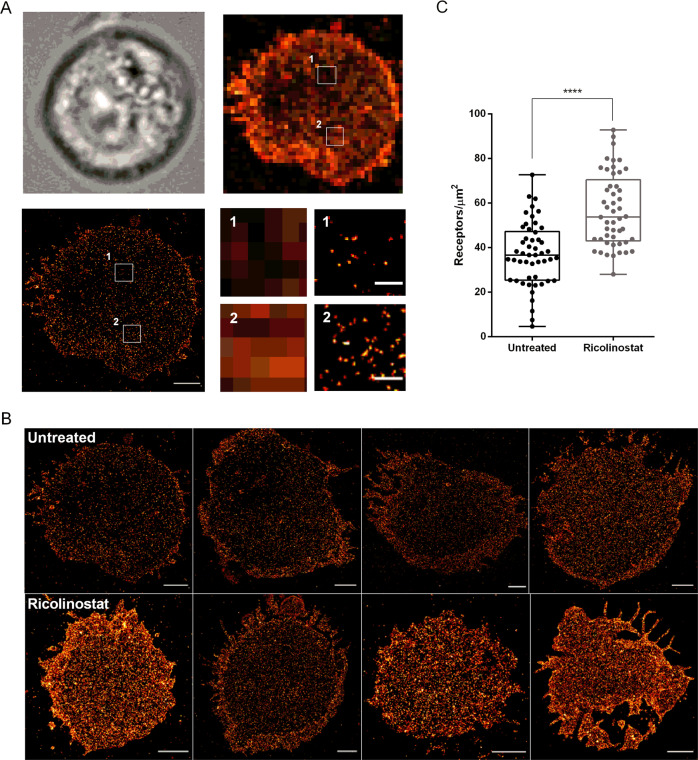
Ricolinostat treatment leads to enhanced CD38 molecule density on MM.1S cells. **a** MM.1S cells were visualized by transmitted light microscopy (upper left). Expression of CD38 was detected by conventional wide-field fluorescence microscopy (upper right) and *d*STORM (lower left). Small panels display magnification of boxed regions revealing the enhanced single-molecule sensitivity of *d*STORM. Scale bars, 2 and 0.2 µm (magnification). **b** Example images of CD38 molecule distribution on untreated and ricolinostat-treated MM.1S cell surface visualized by *d*STORM. Scale bars, 2 µm. **c** Quantification of CD38 molecules (receptors/µm^2^) on ricolinostat-treated (5 µM, *n* = 49 cells) and untreated MM.1S cells (*n* = 50 cells). *p*-Values between indicated groups were calculated using Student’s *t*-test. ****p < 0.0001.

Next, we investigated the density and distribution of CD38 on the surface of MM cells by *d*STORM. We focused on the MM.1S cell line that had the lowest CD38 baseline expression by flow cytometry, and analyzed approximately 50 MM.1S cells by *d*STORM with and without ricolinostat treatment. Single cells were imaged in a time-series for 15,000 frames for ~5 min to reconstruct a super-resolved image with a spatial resolution of ~20 nm (Fig. 2a). *d*STORM images showed that before and after ricolinostat treatment, CD38 molecules were homogeneously distributed across the MM.1S cell membrane without any obvious signs of cluster formation (Fig. 2b). The data demonstrated that CD38 expression was variable between individual MM.1S cells. However, we did not detect CD38 negative MM.1S cells by *d*STORM. Before ricolinostat treatment, the CD38 surface density detected was 5–73 [range] molecules/µm^2^, and increased to 28–93 [range] molecules/µm^2^ after 72 h of treatment with 5 µM ricolinostat (*p* < 0.0001, *n* = 49 cells) (Fig. 2c). In summary, these data confirm that ricolinostat induces increased CD38 surface expression on MM.1S cells. Further, the data suggest that the increase in CD38 expression occurs uniformly and to a similar extent in MM.1S cells that were CD38-high and CD38-low at baseline.

**Fig. 3:**
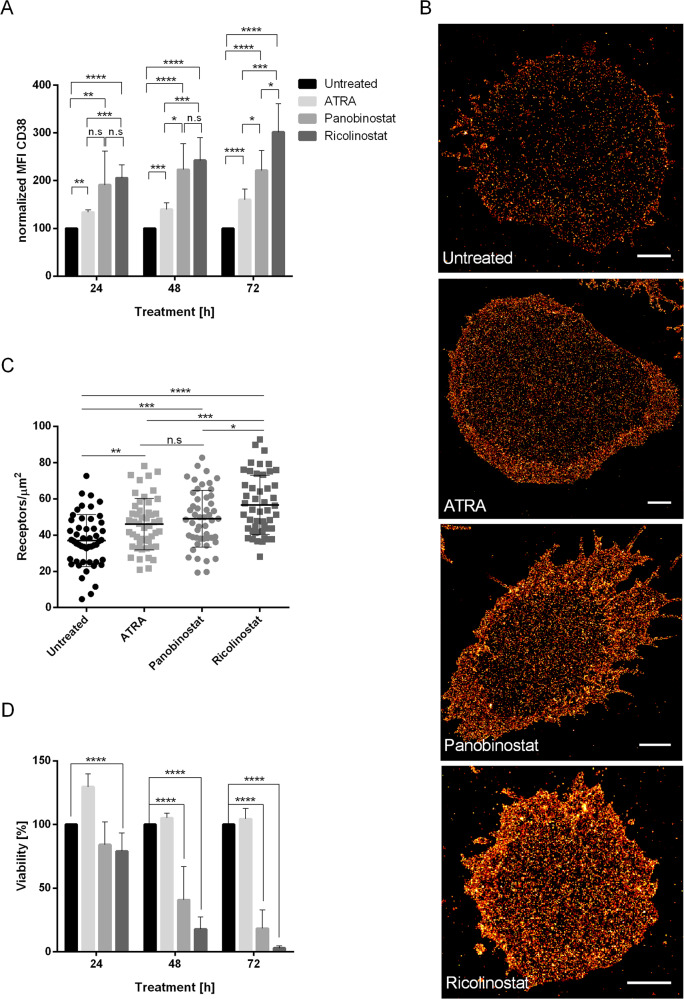
Ricolinostat effect outperforms ATRA and panobinostat induction of CD38 elevation on myeloma cells. MM.1S cells were treated with ATRA and panobinostat at fcs of 10 nM, ricolinostat at a fc of 5 µM or left untreated. **a** Bar diagram shows CD38 expression on MM.1S cells after ATRA (*n* = 5 experiments), panobinostat (*n* = 5 experiments), and ricolinostat (*n* = 5 experiments) treatment as evaluated by flow cytometry. **b** Example images of CD38 molecule distribution on the surface of untreated, ATRA-, panobinostat-, and ricolinostat-treated MM.1S cells visualized by *d*STORM. Scale bars, 2 µm. **c** Quantification of CD38 molecules (receptors/µm^2^) on ATRA- (*n* = 50 cells), panobinostat- (*n* = 50 cells), and ricolinostat-treated (*n* = 49 cells) and untreated MM.1S cells (*n* = 50 cells). **d** Viability of ATRA- (*n* = 5 experiments), panobinostat- (*n* = 5 experiments), and ricolinostat-treated (*n* = 5 experiments) and untreated MM.1S cells (*n* = 5 experiments). Bar diagram shows the percentage of viable (7-AAD neg) MM.1S cells (CD38+/CD138+) determined by flow cytometry. Depicted are mean values with SD. *p*-Values between indicated groups were calculated using Student’s *t*-test. n.s. = not significant, *p < 0.05, **p < 0.005, ***p < 0.001, ****p < 0.0001.

Next, we compared the effect of ricolinostat to CD38 modulators already known; all-trans retinoic acid (ATRA) [26] and panobinostat (panHDAC inhibitor) [16]. We determined CD38 expression on MM.1S by flow cytometry at therapeutic doses. CD38 expression increased after treatment with ATRA, panobinostat, and ricolinostat at all time points. The increase was strongest, however, with ricolinostat. The relative increase in CD38 expression after 72 h of ATRA treatment was 1.6-fold (*p* < 0.0001, *n* = 5), 2.2-fold after panobinostat treatment (*p* < 0.0001, *n* = 5), and 3.1-fold after ricolinostat treatment (*p* < 0.0001, *n* = 5) (Fig. 3a). We verified this observation by *d*STORM, where the CD38 surface density was 21–78 [range] molecules/µm^2^ after treatment with ATRA (*p* < 0.0005, *n* = 50 cells) and 19–82 [range] molecules/µm^2^ after treatment with panobinostat (*p* < 0.001, *n* = 50 cells). Thus, CD38 surface density after ricolinostat treatment was significantly higher with 28–93 [range] molecules/µm^2^ (*p* < 0.0001, *n* = 49 cells) (Fig. 3b, c). Thus, in line with flow cytometry data, ricolinostat treatment produced the greatest increase in CD38 density and in the absolute number of CD38 molecules per MM.1S cell (Fig. 3c). In addition, ricolinostat exerted a more pronounced direct cytotoxic anti-MM effect than panobinostat (Fig. 3d and Supplementary Fig. 3).

Taken together, our data demonstrate that ricolinostat induces increased CD38 surface expression on MM.1S cells and is more potent than the established CD38 modulators panobinostat and ATRA.

### Ricolinostat-induced upregulation of CD38 is specific on myeloma cells

As well as being expressed on MM cells, CD38 marks T-cell activation [27]. We analyzed resting and activated CD8+ and CD4+ T cells at different time points after treatment with ricolinostat but did not detect a significant difference in CD38 expression compared to untreated T cells at the 5 µM dose (Supplementary Fig. 4). Similarly, we studied the effect of ricolinostat on other tumor cell lines but did not observe an increase in CD38 expression after ricolinostat in lymphoma or leukemia cell lines (Supplementary Figs. 5 and 6a, b), indicating that upregulation of CD38 by ricolinostat is a specific effect in MM cells.

### Synergistic anti-myeloma efficacy of ricolinostat and daratumumab

**Fig. 4:**
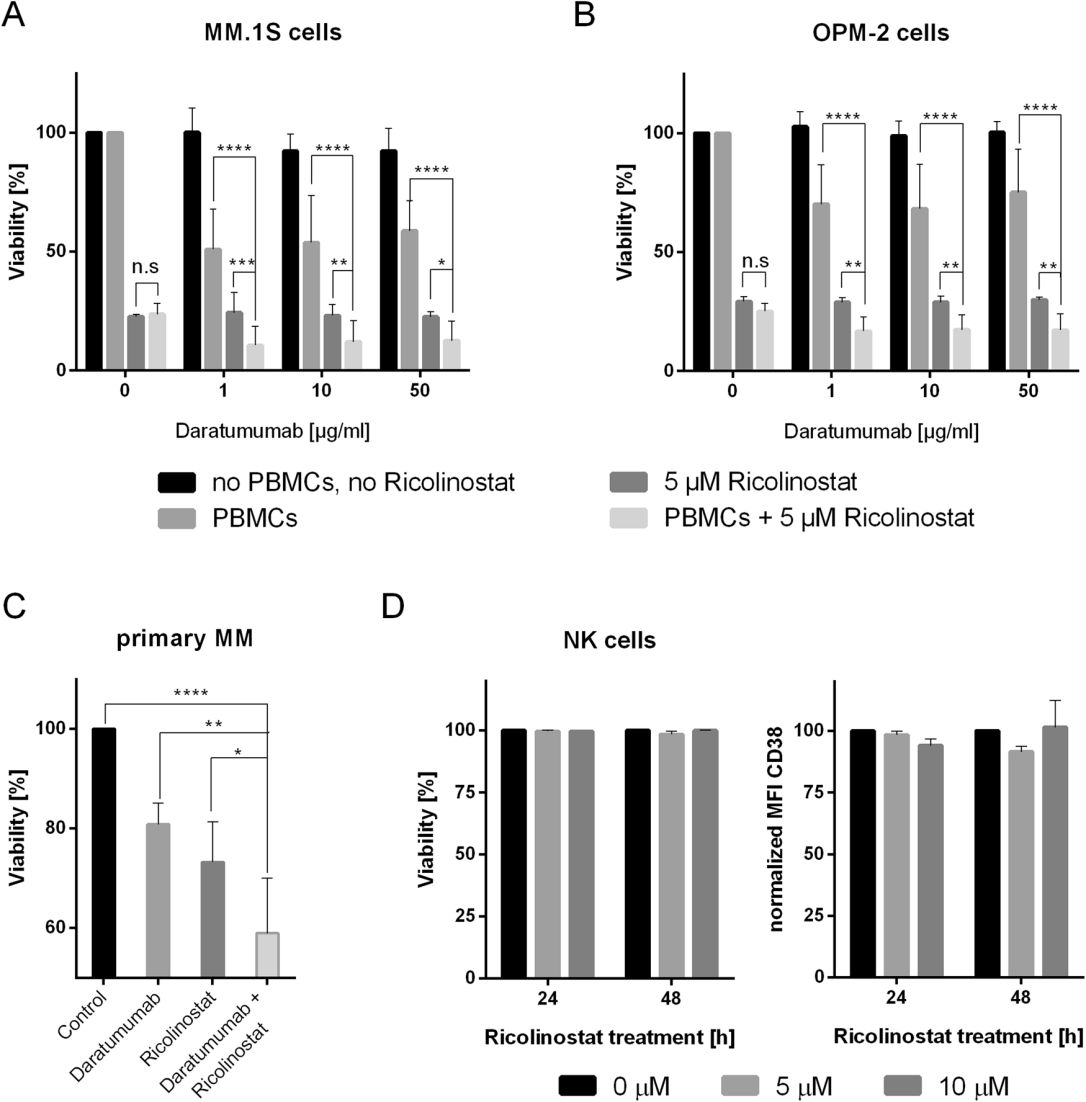
Ricolinostat and daratumumab synergistically eliminate myeloma cells. **a**, **b** ADCC against MM.1S (**a**, *n* = 8 experiments) and OPM-2 (**b**, *n* = 4 experiments) cells with and without ricolinostat treatment. Ricolinostat pre-treatment was performed for 48 h at 5 µM. PBMC from healthy donors (effector-to-target ratio of 25:1) and control antibody or daratumumab were added at the indicated concentrations. Target cells stably express firefly luciferase and viability was analyzed after the addition of luciferin substrate by bioluminescence measurements after 16 h. **c** ADCC of daratumumab against primary MM cells (*n* = 3 patients) with and without ricolinostat treatment. Ricolinostat pre-treatment was performed for 48 h at 5 µM, then autologous PBMCs (effector-to-target ratio of 3:1) and daratumumab (0.1 µg/ml) or control antibody (1 µg/ml) were added to induce ADCC. The percentage of live primary MM cells was determined after 24 h by flow cytometry. The bar diagram shows the percentage of viable (7-AAD neg) CD38+/CD138+ myeloma cells. **d** NK cells were treated with ricolinostat at fcs of 5 and 10 µM for 24 and 48 h and analyzed by flow cytometry (*n* = 2 experiments). Bar diagrams show the percentage of viable (7-AAD neg) cells (left graph) and CD38 expression as normalized MFI of treated vs. untreated cells (right graph). Data are presented as mean values ± SD. *p*-Values between indicated groups were calculated using 2-way ANOVA or Student’s *t*-test. n.s. = not significant, *p < 0.05, **p < 0.005, ***p < 0.001, ****p < 0.0001.

To determine whether the increase in CD38 antigen density enables superior anti-MM activity of the anti-CD38 mAb daratumumab we pretreated MM cell lines with ricolinostat and initiated ADCC with daratumumab or control antibody. Treating MM cell lines with ricolinostat produced increased anti-MM efficacy of ricolinostat and daratumumab. For example, after 16 h, 90% vs. 50% of ricolinostat-treated vs. untreated MM.1S cells were eliminated by daratumumab (*p* < 0.0001, *n* = 8) (Fig. 4a and Supplementary Fig. 7) and 83% vs. 29% of ricolinostat-treated vs. untreated OPM-2 were eliminated by daratumumab (*p* < 0.0001, *n* = 4) (Fig. 4b and Supplementary Fig. 7). When we then incubated primary MM cells from different patients (*n* = 3) with 5 µM of ricolinostat and initiated ADCC with daratumumab or control antibody after 48 h, we also observed a significant increase in ADCC in ricolinostat-treated compared to untreated MM cells. On average, daratumumab eliminated 42% of ricolinostat-treated, but only 19% of untreated, primary MM cells within 24 h (*p* < 0.005, *n* = 3) (Fig. 4c).

Despite the marked increase in CD38 expression after ricolinostat treatment, we were not able to induce CDC using daratumumab against MM cells. Additionally, we studied the expression levels of complement-inhibitory proteins such as CD55 and CD59 before and after ricolinostat treatment and observed an upregulation of CD59, an inhibitor of the terminal pathway of the complement cascade, on primary MM and MM cell lines (Supplementary Fig. 8). Of note, the basal expression level of CD55 on MM.1S cells is similar to Daudi control cell line; however, basal CD59 expression level is dramatically higher in MM.1S compared to Daudi cells, in which we could induce CDC with daratumumab, suggesting that MM.1S and OPM-2 are cell lines resistant to destruction by the complement membrane attack complex (Supplementary Fig. 6c–e).

Next, we analyzed the effect of ricolinostat on NK cells and regulatory T cells. NK cells are considered the main effector cells of ADCC [28, 29], playing a determining role in antibody-mediated cytotoxicity. A subpopulation of regulatory T cells expresses high levels of CD38, demonstrates strong T-cell suppressive capacities, and is effectively depleted by daratumumab [30]. After ricolinostat treatment, there were neither significant changes in viability nor in CD38 expression on both cell populations at any of the dosages tested (Fig. 4d and Supplementary Fig. 9), indicating that ricolinostat does not compromise daratumumab–immune cell interactions via CD38 modulation.

In summary, our data demonstrate that ricolinostat not only exerts a direct anti-MM effect. It also increases CD38 expression on MM cells and thus enhances the anti-MM efficacy of daratumumab through a substantial increase in ADCC.

### Ricolinostat can induce CD38 expression on myeloma cells in daratumumab refractory patients

**Fig. 5:**
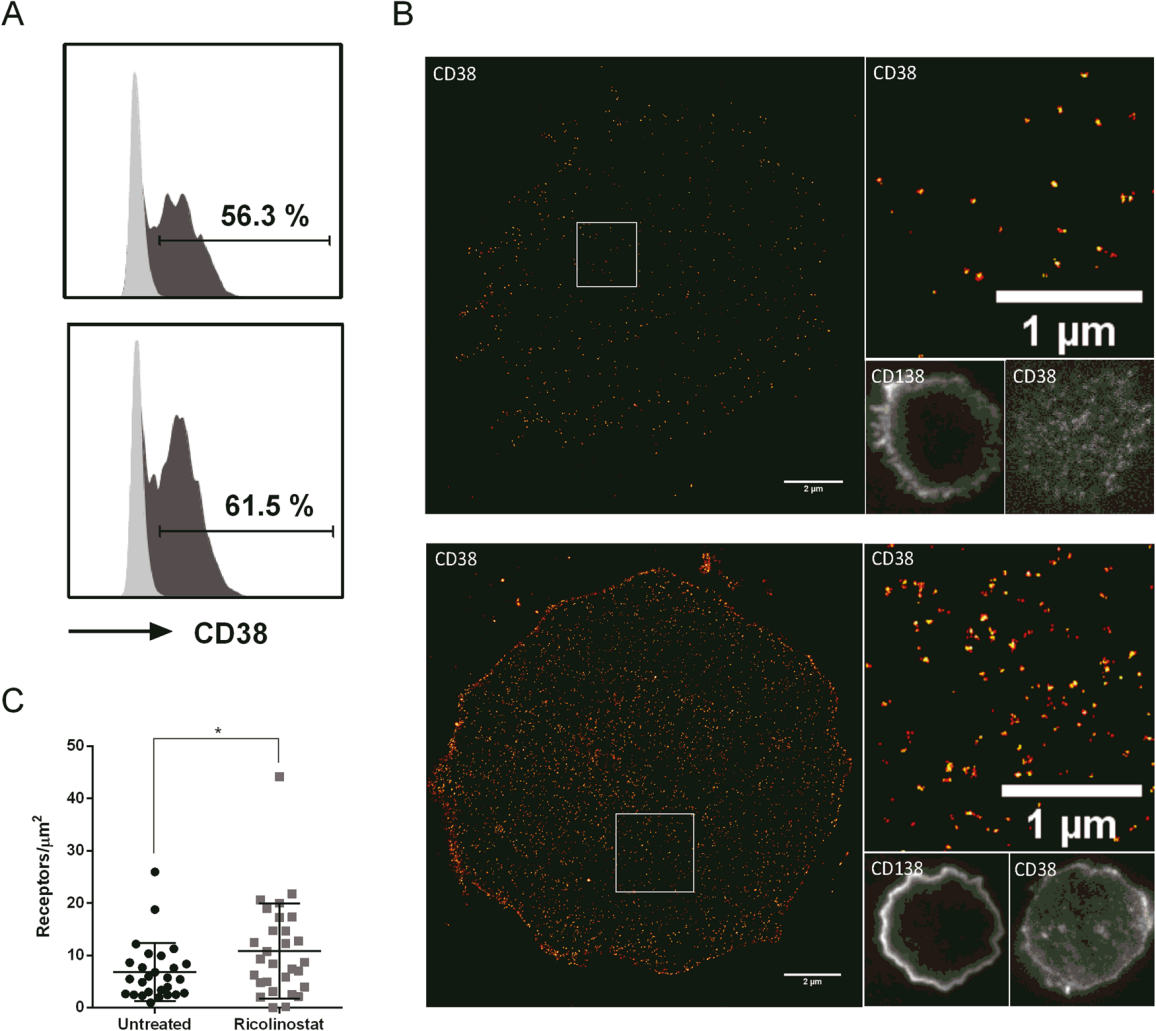
Ricolinostat can induce CD38 expression on myeloma cells in daratumumab refractory patients. **a** The histograms show flow cytometric analysis of CD38 expression on primary MM cells from a daratumumab refractory patient after overnight culture in the absence (upper graph) or presence (lower graph) of 2.5 µM of ricolinostat. **b** Example images of CD38 molecule distribution on the surface of untreated (upper panel) and ricolinostat-treated (2.5 µM) primary MM cells (lower panel) visualized by *d*STORM. **c** Quantification of CD38 molecules (receptors/µm^2^) of ricolinostat-treated (2.5 µM, *n* = 29 cells) and untreated (*n* = 27 cells) primary MM cells. *p*-Values between indicated groups were calculated using Student’s *t*-test. *p < 0.05.

Even if different resistance mechanisms to daratumumab treatment were proposed, CD38 expression is a key determinant of susceptibility to daratumumab [31]. Therefore, we evaluated whether or not ricolinostat enhances CD38 expression on MM cells from patients who have previously undergone daratumumab treatment. First, we analyzed MM cells from an 80 years old patient with IgGλ MM who became refractory after 22 months of daratumumab based therapy (Supplementary Table 1, patient identifier R06). *In vitro* incubation with ricolinostat increased CD38 expression on MM cells as evaluated by flow cytometry from 13.4% at baseline to 24.4% after 24 h and 38.0% after 48 h (corresponding to a 1.5- and 2.3-fold increase in MFI; Supplementary Fig. 10). More importantly, we evaluated MM cells from a 54 years old patient with κLC MM refractory to bortezomib and lenalidomide. We obtained MM cells after the patient had received two cycles of daratumumab but was primarily refractory (Supplementary Table 1, patient identifier R02). After overnight incubation with sub-therapeutic doses of ricolinostat, we detected a mild increase in CD38 expression on MM cells by flow cytometry (Fig. 5a). Using the highly sensitive approach of *d*STORM, we visualized a more than 1.5-fold increase of CD38 surface molecules on MM cells that had escaped treatment with daratumumab (Fig. 5b, c). The increase in CD38 expression as assessed by *d*STORM was statistically significant even with a sub-therapeutic dose of ricolinostat (*p* < 0.05, Fig. 5c). Thus, in both cases, prior failure to respond to daratumumab treatment did not prevent the increase of CD38 expression upon *in vitro* exposure to ricolinostat.

### Upregulation of CD38 expression on multiple myeloma cells by novel HDAC6 inhibitors is a class effect

**Fig. 6:**
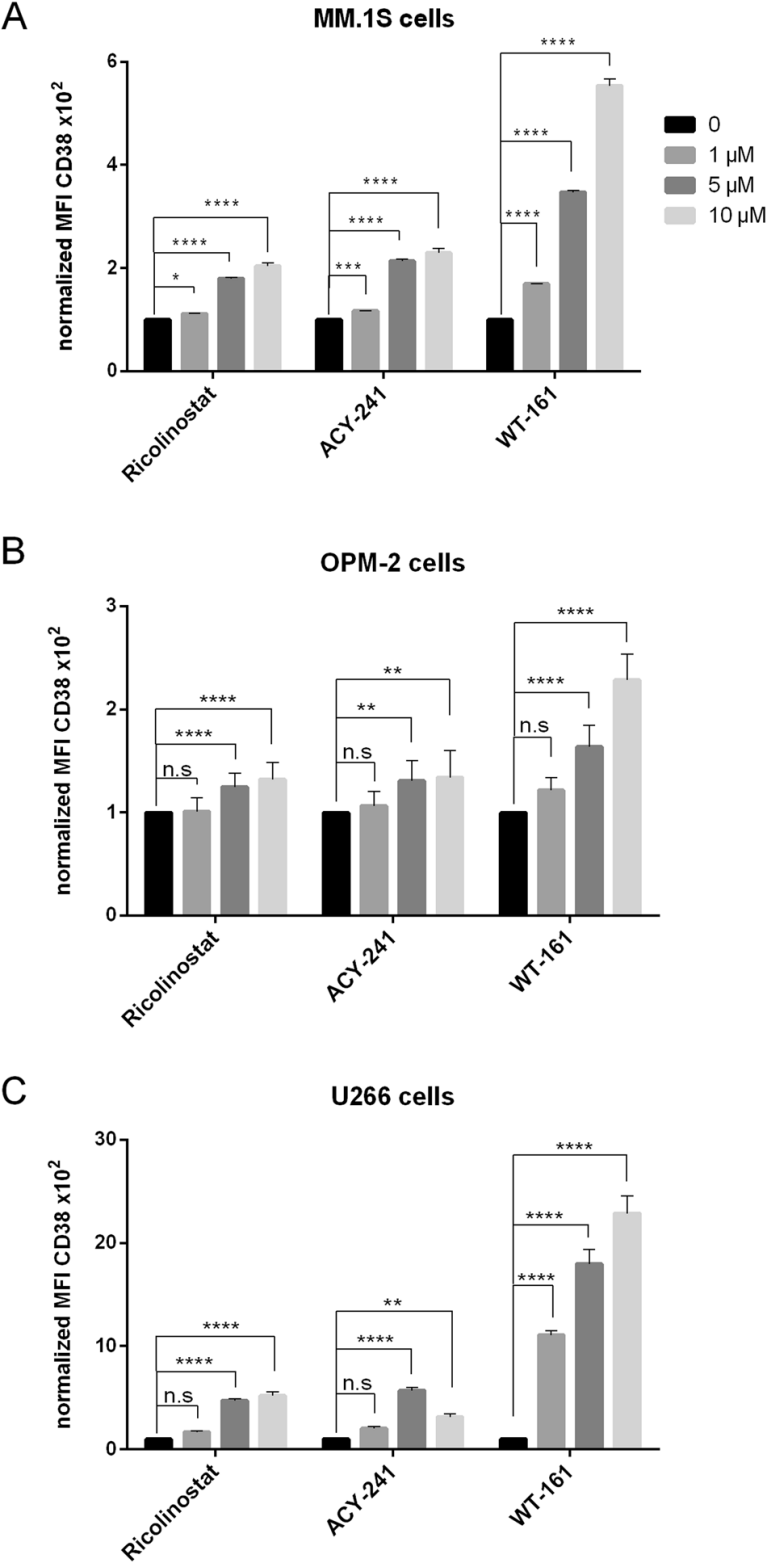
Upregulation of CD38 expression on multiple myeloma cells by novel HDAC6 inhibitors is a class effect. **a**–**c** CD38 expression on MM.1S (**a**, *n* = 6 experiments), OPM2 (**b**, *n* = 6 experiments), and U266 (**c**, *n* = 3 experiments) cells before and after treatment with ricolinostat, ACY-241, and WT-161 at fcs of 1, 5, and 10 µM. Bar diagrams show CD38 expression as normalized MFI of treated vs. untreated cells after 48 h. Depicted are mean values with SD. *p*-Values between indicated groups were calculated using Student’s *t*-test. n.s. = not significant, *p < 0.05, **p < 0.005, ***p < 0.001, ****p < 0.0001.

We also evaluated the effects of the second-generation HDAC6 inhibitors ACY-241 and WT-161 on MM.1S cells and observed a 2-fold increase in CD38 expression by MFI after 48 h of treatment with ACY-241 (*p* < 0.0001, *n* = 3) and 6-fold increase in CD38 expression after treatment with WT-161 (*p* < 0.0001, *n* = 3) (Fig. 6a). Similar trends were obtained for OPM-2 and U266 cells (Fig. 6b, c). ACY-241 and WT-161 also exerted a direct cytotoxic anti-MM effect in the cell lines (Supplementary Fig. 11).

**Fig. 7:**
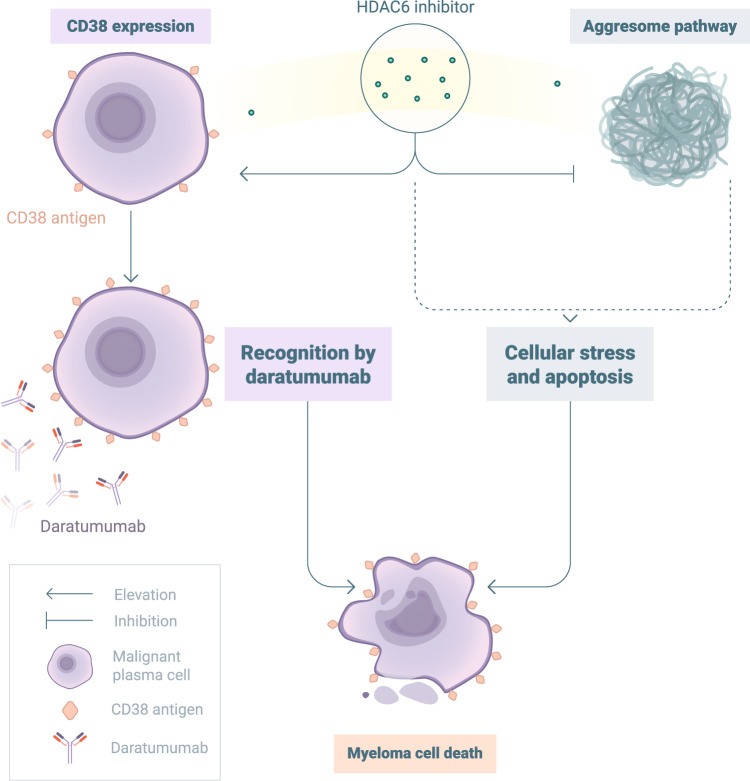
In combination with daratumumab, novel HDAC6 inhibitors exert a dual mode of action against myeloma cells. By disabling the aggresome, HDAC6 inhibitors induce cellular stress and apoptosis of MM cells. In combination regimens, it is possible to exploit the induction of CD38 elevation by HDAC6 inhibitors to enhance the anti-MM efficacy of anti-CD38 mAbs through a substantial increase in ADCC. Therefore, synergistic use of HDAC6 inhibitors and daratumumab is possible to increase response rates and extend response duration in MM patients.

In aggregate, our data show that the second-generation HDAC6 inhibitors ACY-241 and WT-161 potently induce MM cell death and upregulate CD38 expression in a way similar to that of ricolinostat. This suggests that the upregulation of CD38 expression on MM cells by HDAC6 inhibitors is a class effect that can be exploited in combination with CD38-directed therapies (Fig. 7).

## Discussion

Targeted immunotherapy with mAbs has become critical for the successful treatment of many forms of cancer. In MM, CD38-based therapy was developed as an attractive treatment strategy. We recently reported that panobinostat increases the expression of CD38 on MM cells leading to an improvement of daratumumab-mediated ADCC [16]. Here, our research demonstrates that also the novel HDAC6 inhibitor ricolinostat increases CD38 expression specifically on MM cells. We confirm the increase of CD38 molecules after ricolinostat treatment measured by flow cytometry using the highly sensitive *d*STORM approach. The increase of CD38 expression is correlated with higher CD38 RNA levels inside the MM cells. Moreover, ChIP experiments demonstrate that under ricolinostat treatment, histone 3 lysine 27 in the CD38 promoter is more acetylated as a consequence of HDAC6 inhibition by ricolinostat. Therefore, we postulate that in the absence of ricolinostat, HDAC6 deacetylates the CD38 promoter, and during ricolinostat treatment, HDAC6 is inhibited resulting in higher acetylation and, hence, the activation of the CD38 promoter.

ATRA [26] is also able to upregulate CD38 on MM cells as does panobinostat [16]. The increase in CD38 expression after treatment with ricolinostat is significantly higher than after panobinostat or ATRA treatment. For this reason, together with the fact that ricolinostat shows better efficacy and less toxicity than other HDAC inhibitors, ricolinostat appears a promising treatment option to enhance selective tumor antigen expression on MM cells [32].

In line with this observation, we demonstrate that the ricolinostat-induced increase in CD38 expression can be exploited to enhance the anti-MM efficacy of daratumumab through a substantial increase in ADCC. This synergistic effect occurs in both MM cell lines and primary MM cells. Of considerable interest is that ricolinostat alters CD38 expression specifically on MM but not on T cells, including regulatory T cells, or on NK cells. These cell populations are of particular relevance for the control of MM. Tumor antigen-specific T cells have direct anti-MM efficacy [33], and regulatory T cells contribute to the immunosuppressive microenvironment of the MM bone marrow niche. NK cells play a major role in daratumumab-mediated ADCC [28, 29], whereas the role of other effector cells (e.g., neutrophils, γ/δ T cells) remains unclear. Our data suggest that an increase in on-target off-tumor toxicity of daratumumab by ricolinostat is unlikely in T cells and NK cells [32], while regulatory T cells remain susceptible to daratumumab-mediated depletion. It has previously been shown that daratumumab promotes T-cell expansion, T-cell functional response, and TCR clonality in MM [30], and that HDAC inhibitors can deplete myeloid derived suppressor cells, another potent immunosuppressive cell population favoring tumor immune evasion in the MM microenvironment [34]. Together, these findings suggest the possibility of combining ricolinostat and daratumumab in a very effective immunomodulating therapy.

An important clinical observation is that CD38 expression load correlates with anti-MM efficacy of daratumumab. Moreover, pre-clinical work shows that antigen density is a critical variable for daratumumab-mediated ADCC [26]. Our research shows that ricolinostat induces a significant increase in CD38 expression on MM cells from patients with R/R disease previously treated with IMiDs and PIs. However, we also observed increased levels of CD38 expression on primary MM cells that escaped treatment with daratumumab. This was illustrated by the use of *d*STORM to enable ultra-sensitive detection of changes in the CD38 surface density. *d*STORM even detected changes at levels scarcely detectable by flow cytometry that may nevertheless be of potential therapeutic relevance [20]. Therefore, it is entirely possible that treatment with ricolinostat is able to (re)sensitize MM cells to anti-CD38-based-therapies preventing antigen escape, increasing response rates, and extending duration of responses.

Daratumumab has little or no potential to confer complement-dependent cytotoxicity (CDC) against native primary MM cells and many MM cell lines such as MM.1S and OPM-2 [15, 35]. In fact, despite the marked increase in CD38 expression after ricolinostat-treatment, we were unable to induce daratumumab-mediated CDC against these MM cell lines. This inability was possibly also due to the upregulation of the complement inhibitor CD59 on primary MM and MM cell lines. Other effector responses to IgG antibodies include the release of inflammatory mediators and phagocytosis. As daratumumab also acts through antibody-dependent cellular phagocytosis (ADCP) [36], and HDAC6 inhibitors were previously shown to improve antimicrobial human macrophage responses via increased production of reactive oxygen species [37], it is possible that combination therapy can result in enhanced ADCP of MM cells.

In aggregate, on the basis of our data, we anticipate a strong gain of anti-MM efficacy after ricolinostat treatment because ADCC is the leading mode of action of daratumumab [35].

Furthermore, other functional implications of increased CD38 expression should not be disregarded. In this sense, it has also been reported that extramedullary myeloma cells lose the expression of CD38 [38, 39], suggesting that CD38 down-regulation may facilitate the migration of malignant plasma cells to peripheral blood or extramedullary sites. Via interaction with CD31, high levels of CD38 may lead to increased adhesion to stromal cells, maintaining MM cells in the immunosuppressive microenvironment of the bone marrow, but also potentially reducing the risk of extramedullary disease. On the other hand, due to its ectoenzymatic activity, increased CD38 expression could interfere with levels of cyclic adenosine diphosphate (ADP)-ribose and nicotinic acid adenine dinucleotide phosphate (NAADP), two metabolites critical for calcium homeostasis [40], and adenosine that has predominantly immunosuppressive effects on T, NK, and dendritic cells [41]. As daratumumab is a relatively weak inhibitor of ectoenzymatic activity of CD38 [42], implications on the mechanism of action of daratumumab are possible. However, such effects could be reduced by the use of alternative anti-CD38 antibodies with increased enzyme-blocking capacity, such as isatuximab [42].

The second-generation HDAC6 inhibitors ACY-241 and WT-161 potently induce MM cell death in our research and similarly upregulate CD38 expression. This suggests that the upregulation of CD38 expression on MM cells by HDAC6 inhibitors is a class effect.

Therefore, our data provide a rationale for why it is possible to use HDAC6 inhibitors and daratumumab synergistically to increase response rates and extend response duration in MM patients. The data also provide encouraging support for clinical trials to investigate the safety and efficacy of this combination treatment. It has long been known that the use of combination regimens has great potential for improving the efficacy of anti-MM treatment [3]. This research provides a mechanistic approach to reduce the danger of CD38 antigen loss, one of the weaknesses of effective targeted therapy with daratumumab. Thus, our data encourage further efforts to identify and validate HDAC inhibitors, as single agent or in combination, to induce maximal increase in CD38 expression to improve the efficacy of antibody and cellular immunotherapy in MM.

## Supplementary information

Supplementary Information, clean version

Supplemental Table 1

Supplemental Figure 1

Supplemental Figure 2

Supplemental Figure 3

Supplemental Figure 4

Supplemental Figure 5

Supplemental Figure 6

Supplemental Figure 7

Supplemental Figure 8

Supplemental Figure 9

Supplemental Figure 10

Supplemental Figure 11

## References

[1] Kumar SK, Rajkumar SV, Dispenzieri A, Lacy MQ, Hayman SR, Buadi FK (2008). Improved survival in multiple myeloma and the impact of novel therapies. Blood.

[2] Richardson PG, Xie W, Jagannath S, Jakubowiak A, Lonial S, Raje NS (2014). A phase 2 trial of lenalidomide, bortezomib, and dexamethasone in patients with relapsed and relapsed/refractory myeloma. Blood.

[3] Dimopoulos MA, Beksac M, Benboubker L, Roddie H, Allietta N, Broer E (2013). Phase II study of bortezomib-dexamethasone alone or with added cyclophosphamide or lenalidomide for sub-optimal response as second-line treatment for patients with multiple myeloma. Haematologica.

[4] Ayed AO, Chang LJ, Moreb JS (2015). Immunotherapy for multiple myeloma: current status and future directions. Crit Rev Oncol/Hematol.

[5] Kumar SK, Lee JH, Lahuerta JJ, Morgan G, Richardson PG, Crowley J (2012). Risk of progression and survival in multiple myeloma relapsing after therapy with IMiDs and bortezomib: a multicenter international myeloma working group study. Leukemia.

[6] van de Donk NW, Moreau P, Plesner T, Palumbo A, Gay F, Laubach JP (2016). Clinical efficacy and management of monoclonal antibodies targeting CD38 and SLAMF7 in multiple myeloma. Blood.

[7] Lonial S, Weiss BM, Usmani SZ, Singhal S, Chari A, Bahlis NJ (2016). Daratumumab monotherapy in patients with treatment-refractory multiple myeloma (SIRIUS): an open-label, randomised, phase 2 trial. Lancet.

[8] Malavasi F, Deaglio S, Funaro A, Ferrero E, Horenstein AL, Ortolan E (2008). Evolution and function of the ADP ribosyl cyclase/CD38 gene family in physiology and pathology. Physiol Rev.

[9] Malavasi F (2011). Editorial: CD38 and retinoids: a step toward a cure. J Leukoc Biol.

[10] Vaisitti T, Aydin S, Rossi D, Cottino F, Bergui L, D’Arena G (2010). CD38 increases CXCL12-mediated signals and homing of chronic lymphocytic leukemia cells. Leukemia.

[11] Usmani SZ, Weiss BM, Plesner T, Bahlis NJ, Belch A, Lonial S (2016). Clinical efficacy of daratumumab monotherapy in patients with heavily pretreated relapsed or refractory multiple myeloma. Blood.

[12] Palumbo A, Chanan-Khan A, Weisel K, Nooka AK, Masszi T, Beksac M (2016). Daratumumab, bortezomib, and dexamethasone for multiple myeloma. N Engl J Med.

[13] Dimopoulos MA, Oriol A, Nahi H, San-Miguel J, Bahlis NJ, Usmani SZ (2016). Daratumumab, lenalidomide, and dexamethasone for multiple myeloma. N Engl J Med.

[14] Chari A, Suvannasankha A, Fay JW, Arnulf B, Kaufman JL, Ifthikharuddin JJ (2017). Daratumumab plus pomalidomide and dexamethasone in relapsed and/or refractory multiple myeloma. Blood.

[15] Nijhof IS, Casneuf T, van Velzen J, van Kessel B, Axel AE, Syed K (2016). CD38 expression and complement inhibitors affect response and resistance to daratumumab therapy in myeloma. Blood.

[16] Garcia-Guerrero E, Gogishvili T, Danhof S, Schreder M, Pallaud C, Perez-Simon JA (2017). Panobinostat induces CD38 upregulation and augments the antimyeloma efficacy of daratumumab. Blood.

[17] Vogl DT, Raje N, Jagannath S, Richardson P, Hari P, Orlowski R (2017). Ricolinostat, the first selective histone deacetylase 6 inhibitor, in combination with bortezomib and dexamethasone for relapsed or refractory multiple myeloma. Clin Cancer Res.

[18] Gao X, Shen L, Li X, Liu J (2019). Efficacy and toxicity of histone deacetylase inhibitors in relapsed/refractory multiple myeloma: systematic review and meta-analysis of clinical trials. Exp Ther Med.

[19] van de Linde S, Loschberger A, Klein T, Heidbreder M, Wolter S, Heilemann M (2011). Direct stochastic optical reconstruction microscopy with standard fluorescent probes. Nat Protoc.

[20] Nerreter T, Letschert S, Gotz R, Doose S, Danhof S, Einsele H (2019). Super-resolution microscopy reveals ultra-low CD19 expression on myeloma cells that triggers elimination by CD19 CAR-T. Nat Commun.

[21] Heilemann M, van de Linde S, Schuttpelz M, Kasper R, Seefeldt B, Mukherjee A (2008). Subdiffraction-resolution fluorescence imaging with conventional fluorescent probes. Angew Chem.

[22] Carpenter RO, Evbuomwan MO, Pittaluga S, Rose JJ, Raffeld M, Yang S (2013). B-cell maturation antigen is a promising target for adoptive T-cell therapy of multiple myeloma. Clin Cancer Res.

[23] Seckinger A, Delgado JA, Moser S, Moreno L, Neuber B, Grab A (2017). Target expression, generation, preclinical activity, and pharmacokinetics of the BCMA-T cell bispecific antibody EM801 for multiple myeloma treatment. Cancer Cell.

[24] Lonial S, Dimopoulos M, Palumbo A, White D, Grosicki S, Spicka I (2015). Elotuzumab therapy for relapsed or refractory multiple myeloma. N Engl J Med.

[25] Santo L, Hideshima T, Kung AL, Tseng JC, Tamang D, Yang M (2012). Preclinical activity, pharmacodynamic, and pharmacokinetic properties of a selective HDAC6 inhibitor, ACY-1215, in combination with bortezomib in multiple myeloma. Blood.

[26] Nijhof IS, Groen RW, Lokhorst HM, van Kessel B, Bloem AC, van Velzen J (2015). Upregulation of CD38 expression on multiple myeloma cells by all-trans retinoic acid improves the efficacy of daratumumab. Leukemia.

[27] Mehta K, Shahid U, Malavasi F (1996). Human CD38, a cell-surface protein with multiple functions. FASEB J.

[28] Mahaweni NM, Bos GMJ, Mitsiades CS, Tilanus MGJ, Wieten L (2018). Daratumumab augments alloreactive natural killer cell cytotoxicity towards CD38+ multiple myeloma cell lines in a biochemical context mimicking tumour microenvironment conditions. Cancer Immunol Immunother.

[29] van de Donk NW, Janmaat ML, Mutis T, Lammerts van Bueren JJ, Ahmadi T, Sasser AK (2016). Monoclonal antibodies targeting CD38 in hematological malignancies and beyond. Immunol Rev.

[30] Krejcik J, Casneuf T, Nijhof IS, Verbist B, Bald J, Plesner T (2016). Daratumumab depletes CD38+ immune regulatory cells, promotes T-cell expansion, and skews T-cell repertoire in multiple myeloma. Blood.

[31] van de Donk N, Usmani SZ (2018). CD38 antibodies in multiple myeloma: mechanisms of action and modes of resistance. Front Immunol.

[32] Cengiz Seval G, Beksac M (2019). A comparative safety review of histone deacetylase inhibitors for the treatment of myeloma. Expert Opin Drug Saf.

[33] Bae J, Hideshima T, Zhang GL, Zhou J, Keskin DB, Munshi NC (2018). Identification and characterization of HLA-A24-specific XBP1, CD138 (Syndecan-1) and CS1 (SLAMF7) peptides inducing antigens-specific memory cytotoxic T lymphocytes targeting multiple myeloma. Leukemia.

[34] Wang HF, Ning F, Liu ZC, Wu L, Li ZQ, Qi YF (2017). Histone deacetylase inhibitors deplete myeloid-derived suppressor cells induced by 4T1 mammary tumors in vivo and in vitro. Cancer Immunol Immunother.

[35] de Weers M, Tai YT, van der Veer MS, Bakker JM, Vink T, Jacobs DC (2011). Daratumumab, a novel therapeutic human CD38 monoclonal antibody, induces killing of multiple myeloma and other hematological tumors. J Immunol.

[36] Overdijk MB, Verploegen S, Bogels M, van Egmond M, Lammerts van Bueren JJ, Mutis T (2015). Antibody-mediated phagocytosis contributes to the anti-tumor activity of the therapeutic antibody daratumumab in lymphoma and multiple myeloma. mAbs.

[37] Ariffin JK, das Gupta K, Kapetanovic R, Iyer A, Reid RC, Fairlie DP (2015). Histone deacetylase inhibitors promote mitochondrial reactive oxygen species production and bacterial clearance by human macrophages. Antimicrob Agents Chemother.

[38] Costa F, Dalla Palma B, Giuliani N (2019). CD38 expression by myeloma cells and its role in the context of bone marrow microenvironment: modulation by therapeutic agents. Cells.

[39] Tembhare P, Yuan C, Korde N, Maric I, Landgren O (2012). Antigenic drift in relapsed extramedullary multiple myeloma: plasma cells without CD38 expression. Leuk Lymphoma.

[40] Howard M, Grimaldi JC, Bazan JF, Lund FE, Santos-Argumedo L, Parkhouse RM (1993). Formation and hydrolysis of cyclic ADP-ribose catalyzed by lymphocyte antigen CD38. Science.

[41] Chillemi A, Quarona V, Antonioli L, Ferrari D, Horenstein AL, Malavasi F (2017). Roles and modalities of ectonucleotidases in remodeling the multiple myeloma niche. Front Immunol.

[42] van Bueren JL, Jakobs D, Kaldenhoven N, Roza M, Hiddingh S, Meesters J (2014). Direct in vitro comparison of daratumumab with surrogate analogs of CD38 antibodies MOR03087, SAR650984 and Ab79. Blood.

