# Publisher Correction: Upregulation of CD38 expression on multiple myeloma cells by novel HDAC6 inhibitors is a class effect and augments the efficacy of daratumumab

**DOI:** 10.1038/s41375-021-01355-6

**Published:** 2021-11-29

**Authors:** Estefanía García-Guerrero, Ralph Götz, Sören Doose, Markus Sauer, Alfonso Rodríguez-Gil, Thomas Nerreter, K. Martin Kortüm, José A. Pérez-Simón, Hermann Einsele, Michael Hudecek, Sophia Danhof

**Affiliations:** 1 Instituto de Biomedicina de Sevilla (IBIS), Hospital Universitario Virgen del Rocío/CSIC/Universidad de Sevilla, Sevilla, Spain; 2 Department of Biotechnology and Biophysics, Biocenter, and RVZ for Integrative and Translational BioImaging, Julius-Maximilians-Universität Würzburg, Würzburg, Germany; 3 Medizinische Klinik und Poliklinik II, Universitätsklinikum Würzburg, Würzburg, Germany

**Keywords:** Combination drug therapy, Myeloma, Immunotherapy

Correction to: *Leukemia* 10.1038/s41375-020-0840-y, published online 29 April 2020

The article Upregulation of CD38 expression on multiple myeloma cells by novel HDAC6 inhibitors is a class effect and augments the efficacy of daratumumab, written by Estefanía García-Guerrero, Ralph Götz, Sören Doose, Markus Sauer, Alfonso Rodríguez-Gil, Thomas Nerreter, K. Martin Kortüm, José A. Pérez-Simón, Hermann Einsele, Michael Hudecek & Sophia Danhof, was originally published Online First without Open Access. After publication in volume 35, page 201–214, the author decided to opt for Open Choice and to make the article an Open Access publication. Therefore, the copyright of the article has been changed to © The Author(s) 2021 and the article is forthwith distributed under the terms of the Creative Commons Attribution.

