# Supplementary material for: Upregulation of CD38 expression on multiple myeloma cells by novel HDAC6 inhibitors is a class effect and augments the efficacy of daratumumab
Source: Leukemia. 2020 Apr 29;35(1):201–14. doi: 10.1038/s41375-020-0840-y (PMC8318885; doi:10.1038/s41375-020-0840-y)
Supplement: Supplementary file 1 — Supplementary Information, clean version [file 41375_2020_840_MOESM1_ESM.docx]

**Supplementary Information**

**Upregulation of CD38 Expression on Multiple Myeloma Cells by Novel HDAC6 Inhibitors is a Class Effect and Augments the Efficacy of Daratumumab**

*Short title: Upregulation of CD38 on Myeloma Cells by Novel HDAC6 Inhibitors*

Estefanía García-Guerrero^1^, Ralph Götz^2^, Sören Doose^2^, Markus Sauer^2^, Alfonso Rodriguez-Gil^1^, Thomas Nerreter^3^, K. Martin Kortüm^3^, José A. Pérez-Simón^1^, Hermann Einsele^3^, Michael Hudecek^3^ and Sophia Danhof^3^

^1^Instituto de Biomedicina de Sevilla (IBIS), Hospital Universitario Virgen del Rocío/CSIC/Universidad de Sevilla, Sevilla, Spain; ^2^ Department of Biotechnology and Biophysics, Biocenter, Julius-Maximilians-Universität Würzburg, Am Hubland, Würzburg, Germany; ^3^Medizinische Klinik und Poliklinik II, Universitätsklinikum Würzburg, Würzburg, Germany

Correspondence to:

Sophia Danhof

Medizinische Klinik und Poliklinik II

Universitätsklinikum Würzburg

Oberdürrbacher Strasse 6

97080 Würzburg, Germany

Email: Danhof_S@ukw.de

Phone: +49 931 201-71091

Fax: +49 931 201-671091

**Key words:**

Multiple myeloma,

Cancer immunotherapy,

HDAC inhibitors,

CD38,

*d*STORM

**Supplementary Information**

**Supplementary Materials and Methods**

**Cell lines**

The MM.1S, OPM-2 and U266 (multiple myeloma, MM), Raji and Daudi (burkitt lymphoma), JeKo-1 (mantle cell lymphoma) and Jurkat (acute T-cell leukemia) cell lines were purchased from DSMZ (Braunschweig, Germany). Contamination of mycoplasma was regularly examined by PCR, and no contamination was detected during experiments concerning this work. MM.1S/ffluc and OPM-2/ffluc were derived by lentiviral transduction with the *Firefly luciferase (ffluc)* gene.

**Antibodies**

| **Name of antibody** | **Vendor** | **Catalog number** |
| --- | --- | --- |
| anti-CD38 | BioLegend (London, UK) | 303526 |
| anti-CD38 | BioLegend (London, UK) | 303512 |
| anti-CD138 | BioLegend (London, UK) | 356508 |
| anti-CD8 | BioLegend (London, UK) | 344722 |
| anti-CD4 | BioLegend (London, UK) | 300552 |
| anti-CD55 | BioLegend (London, UK) | 311315 |
| anti-CD59 | BioLegend (London, UK) | 304707 |
| anti-CD319 (SLAMF7) | BioLegend (London, UK) | 331806 |
| anti-CD269 (BCMA) | BioLegend (London, UK) | 357506 |
| anti-CD19 | BD Biosciences (San Jose, CA) | 641395 |
| anti-CD25 | BD Biosciences (San Jose, CA) | 347643 |
| anti-CD25 | BD Biosciences (San Jose, CA) | 340939 |
| anti-CD127 | BD Biosciences (San Jose, CA) | 557938 |
| anti-CD3 | BD Biosciences (San Jose, CA) | 345767 |
| anti-CD56 | BD Biosciences (San Jose, CA) | 345810 |
| anti-CD45 | BD Horizon (Franklin Lakes, NJ) | 642275 |
| 7-AAD | BD Pharmingen (San Diego, CA) | 559925 |

**Quantitation of CD38 mRNA levels**

Total RNAs were extracted with RNeasy Mini Kit (Qiagen, Hilden, Germany) according to the manufacturer's protocol. Reverse transcription‑quantitative polymerase chain reaction (RT-qPCR) analysis of CD38 was performed with 1 µg of total RNA and SuperScript™ II Reverse Transcriptase (Thermo Fisher Scientific, Inc Massachusetts). The quality and integrity of the RNA was verified by a Bioanalyzer 2100 (Agilent Technologies, Santa Clara, CA). The primer sequences used were as follows: CD38 forward primer, 5'- CTT TCC CGA GAC CGT CCT G-3' and reverse primer, 5'- TGC ACC CTT GAA AGC ATC CC-3'. Primers specific for β-actin were used as a control (forward, 5'-TCC ATC ATG AAG TGT GAC GT-3' and reverse, 5'-GAG CAA TGATCTTGATCT TCA T-3'). RT-qPCR was performed in a 7900HT Real-time PCR System (Thermo Fisher Scientific, Inc Massachusetts) using Quantitec SYBR green Kit (Qiagen, Hilden, Germany) in a 7900 HT Fast Real Time PCR System (Applied Biosystems, Foster City, CA). PCR conditions consisted of the following: 95˚C for 3 min for denaturation; 95˚C for 30 sec for annealing; and 62˚C for 40 sec for extension, for 40 cycles. The threshold cycle for each sample was selected from the linear range and converted to a starting quantity by interpolation from a standard curve generated on the same plate for each set of primers. The CD38 messenger (m) RNA levels were normalized for each well to the β-actin mRNA levels using the 2-ΔΔCq method (Livak *et al*. Methods, 2001).

**Chromatin immunoprecipitation assay**

Chromatin immunoprecipitation (ChIP) was performed as follows: MM cells were harvested after 24 h of treatment with Ricolinostat (5 µM) or not treated. Dead cells were eliminated by ficoll gradient purification. Cells were incubated in 1% formaldehyde in PBS for 10 min at room temperature (RT), glycine was added to a final concentration of 0.125 M and incubated 5 min at RT. Cells were centrifuged at 800g, 4°C for 5 min, and washed three times with ice cold PBS. Cell pellets were resuspended in 0,5 ml ChIP lysis buffer (TrisHCl 50 mM, pH 7.5, EDTA 10 mM, SDS 1%, cOmplete™ Protease Inhibitor Cocktail, Roche, Basel, Switzerland), vortexed and incubated for 20 min in ice. Chromatin was sheared by sonicating in a Bioruptor sonicator (30 min in 30sec on-30sec off cycles, power high) to an average size of 500 bp. 20mg of chromatin were diluted 1:10 with ChIP dilution buffer (0.01% (w/v) SDS, 1% (v/v) Triton X-100, 1.2 mMEDTA (pH 8.0), 16.7 mM Tris/HCl (pH 8.0), 167 mM NaCl) and incubated overnight rotating at 4°C with 4 µg of antiH3K27Ac (Active Motif #39133) and 20 µl of Pierce Protein A/G Agarose (Thermo Fisher Scientific, Inc Massachusetts). Beads were centrifuged at 2000g 10 min, 4°C, and washed twice with 1ml Low Salt Buffer (0.1% (w/v) SDS , 1% (v/v) Triton X-100, 2 mM EDTA (pH 8.0), 20 mM Tris/HCl (pH 8.0), 150 mM NaCl, cOmplete™ Protease Inhibitor), twice with High Salt Buffer ((0.1% (w/v) SDS , 1% (v/v) Triton X-100, 2 mM EDTA (pH 8.0), 20 mM Tris/HCl (pH 8.0), 500 mM NaCl, cOmplete™ Protease Inhibitor), and once with TE. Chromatin was eluted by suspending the beads with 100 µl of ChIP elution buffer and incubating 30 min at 65°C with agitation. From this point, 20 µg of chromatin were treated in parallel to the IP samples as input. Samples were treated with 50 μg/ml RNase A for 30 min at 37°C and then crosslink was reversed incubating 16 h at 65°C. Samples were treated with 0.5 μg/μl Proteinase K for 10 min at 56°C, and DNA was purified using a DNeasy Blood and Tissue extraction mini kit (Qiagen, Hilden, Germany). DNA was quantified by RT-qPCR using primers for Actin (ACTB-Prom-F: AAAGGCAACTTTCGGAACGG, ACTB-Prom-R: TTCCTCAATCTCGCTCTCGC) , CD55 (CD55-F: CCACGAGGCTTCTGCTTACT, CD55-R: CCGGGTTAGAACAAGGACGC) and CD38 promoters. Three different primer pairs were used for CD38 promoter (CD38-A-F: GATCCTCGTCGTGGTGCTC, CD38-A-R: TCAGTGTACTTGACGCATCG; CD38-B-F: GCTGTCTCTGACCCGAAAGT, CD38-B-R: CGTGGTTTGGCCCTTCTACA; CD38-C-F: TATGGCCAACTGCGAGTTCA, CD38-C-R: GCCAACCCACCTCATCTCAG). Fold enrichment was calculated normalizing to the input, the Actin promoter and the not treated control signals.

**Quantitative analysis with *direct* Stochastic Optical Reconstruction Microscopy (*d*STORM)**

The myeloma cells were stained for 30 min on ice with anti-CD38-AF647 or -BV421, anti-CD138-FITC (all BioLegend, San Diego, CA) antibodies or isotype controls (5 µg/ml) and fixed overnight at 4°C in 4% PFA after three washing steps. *d*STORM images were taken using an Olympus IX-71 inverted microscope with an oil-immersion objective (Olympus APON 60xO TIRF, NA 1.49) and a nosepiece stage (IX2-NPS, Olympus) to minimize axial drift. Alexa Fluor 647 (AF647) was excited by a 639 nm laser (Genesis MX639-1000, Coherent) with ~ 7 kW/cm² in TIRF-mode and the emission was separated from the excitation laser by a polychromatic mirror (HC 410/504/582/669, Semrock, Rochester, NY, USA). In addition, the emission was filtered by a bandpass filter (HC 679/41, Semrock). The acquisition of 15,000 frames with an integration time of 20 ms was performed using an EMCCD camera (iXon Ultra 897, Andor) with a pixel size of 128 nm in photoswitching buffer (100 mM ß-mercaptoethylamin, pH 7.4). Images were reconstructed with the software rapidSTORM 3.3 (Wolter et al. Nat Methods. 2012) with a fixed PSF model of 360 nm. We quantified the CD38 localization data using a custom script written with Mathematica (Wolfram, UK; Version 11.1).

**Antibody-dependent cellular cytotoxicity (ADCC) assay with myeloma cell lines**

MM cell lines transduced with *Firefly luciferase* *(ffluc)* were treated with 5 µM ricolinostat. After 48h, PBMCs were added at an effector-to-target ratio of 25:1 in triplicate wells of 96-well flat-bottom plates with solvent controls, IgG1 isotype controls or daratumumab. ADCC was determined at 16h in a bioluminescence-based assay (Brown et al. J Immunol Methods, 2005). Bioluminescence was measured after addition of D-Luciferin substrate (OZ Biosciences, Marseilles, France) at a final concentration of 150 μg/ml using a bioluminometer (Tecan, Männedorf, Switzerland). The percentage of viable cells was calculated using the following formula: % viability = mean bioluminescence signal in the presence of effector cells and daratumumab (with or without ricolinostat pre-treatment) × 100 / mean bioluminescence signal in the control condition.

**Complement-dependent cytotoxicity (CDC)**

Primary MM cells and MM cell lines were distributed at a concentration of 1x10e5 cells per well in 96-well flat-bottom plates and treated with ricolinostat at 5 µM. After 48 hours, cells were cultured in RPMI-1640 supplemented with pooled (5 donors) fresh (non-heat inactivated) human serum or heat-inactivated (30 min at 56°C) human serum, and either solvent control, IgG1 isotype control antibody or daratumumab at 37 °C. Cell viability of primary MM cells was determined by flow cytometry after 24 hours using 7-AAD staining. Cell viability of MM cell lines was determined in a bioluminescence-based cytotoxicity assay (Brown et al. J Immunol Methods, 2005) at 16 hours using a bioluminometer. Bioluminescence was measured after addition of D-Luciferin substrate (OZ Biosciences, Marseille, France) to the assay medium to a final concentration of 150 μg/ml. The percentage of viable cells was calculated using the following formula: % viability = mean bioluminescence signal in the presence of fresh human serum and daratumumab (with or without ricolinostat pre-treatment) x 100 / mean bioluminescence signal in the control condition (Brown et al. J Immunol Methods, 2005).

**Supplemental References**

Wolter S, Löschberger A, Holm T, Aufmkolk S, Dabauvalle MC, van de Linde S, Sauer M. rapidSTORM: accurate, fast open-source software for localization microscopy. Nat Methods. 2012 Nov;9(11):1040-1. doi: 10.1038/nmeth.2224.

[Livak KJ](https://www.ncbi.nlm.nih.gov/pubmed/?term=Livak%20KJ%5BAuthor%5D&cauthor=true&cauthor_uid=11846609), [Schmittgen TD](https://www.ncbi.nlm.nih.gov/pubmed/?term=Schmittgen%20TD%5BAuthor%5D&cauthor=true&cauthor_uid=11846609). Analysis of relative gene expression data using real-time quantitative PCR and the 2(-Delta Delta C(T)) Method. Methods 2001; **25**(4):402-8. doi: [10.1006/meth.2001.1262](https://doi.org/10.1006/meth.2001.1262)

[Brown CE](https://www.ncbi.nlm.nih.gov/pubmed/?term=Brown%20CE%5BAuthor%5D&cauthor=true&cauthor_uid=15777929), [Wright CL](https://www.ncbi.nlm.nih.gov/pubmed/?term=Wright%20CL%5BAuthor%5D&cauthor=true&cauthor_uid=15777929), [Naranjo A](https://www.ncbi.nlm.nih.gov/pubmed/?term=Naranjo%20A%5BAuthor%5D&cauthor=true&cauthor_uid=15777929), [Vishwanath RP](https://www.ncbi.nlm.nih.gov/pubmed/?term=Vishwanath%20RP%5BAuthor%5D&cauthor=true&cauthor_uid=15777929), [Chang WC](https://www.ncbi.nlm.nih.gov/pubmed/?term=Chang%20WC%5BAuthor%5D&cauthor=true&cauthor_uid=15777929), [Olivares S](https://www.ncbi.nlm.nih.gov/pubmed/?term=Olivares%20S%5BAuthor%5D&cauthor=true&cauthor_uid=15777929),et al. Biophotonic cytotoxicity assay for high-throughput screening of cytolytic killing. [J Immunol Method](https://www.ncbi.nlm.nih.gov/pubmed/15777929)s 2005; **297**(1-2):39-52. doi: [10.1016/j.jim.2004.11.021](https://doi.org/10.1016/j.jim.2004.11.021)

**Supplementary Figure Legends**

**Supplementary Figure 1. Ricolinostat treatment leads to enhanced CD38-expression on OPM-2 cells.**

Bar diagram shows CD38 expression on OPM-2 cells (n=6 experiments) before and after ricolinostat treatment. The overlay histogram shows flow cytometric analysis of CD38-expression on OPM-2 cells cultured in the absence or presence of 5 µM and 10 µM of ricolinostat, respectively, for 72 hours. Data are presented as mean values ± SD. P-values between indicated groups were calculated using Student´s t-test. *p<.05, **p<.005, ***p<.001

**Supplementary Figure 2. Ricolinostat effect on BCMA and SLAMF7 targets.**

A-C) Bar diagram shows BCMA expression on MM.1S (A, n=7 experiments), OPM-2 (B, n=5 experiments) and primary MM cells (C, n=5 experiments) before and after ricolinostat treatment at 5 µM and 10 µM for 24, 48 and 72 hours.

D-E) Bar diagram shows SLAMF7 expression on MM.1S (D, n=5 experiments) and OPM-2 (E, n=3 experiments) before and after ricolinostat treatment at 5 µM and 10 µM for 24, 48 and 72 hours.

Depicted are mean values ± SD. P-values between indicated groups were calculated using Student´s t-test. n.s = not significant, *p<.05, **p<.005, ***p<.001, ****p<.0001

**Supplementary Figure 3. Cytotoxic anti-myeloma effect of ricolinostat.**

A-C) Viability of MM.1S (A, n=9 experiments), OPM-2 (B, n=5 experiments) and primary MM (C, n=9 patients) cells prior to and after ricolinostat treatment at 5 µM and 10 µM, respectively, for 24, 48 and 72 hours.

D) Cytotoxic effect of ricolinostat on primary MM cells from newly diagnosed (ND, n=4) and relapsed/refractory (R/R, n=5) patients. The bar diagram shows the percentage of viable (7-AAD neg) MM cells (CD38+/CD138+) determined by flow cytometry after exposure to 5 µM of ricolinostat for 48 hours.

Depicted are mean values ± SD. P-values between indicated groups were calculated using Student´s t-test. n.s = not significant, *p<.05, ***p<.001, ****p<.0001

**Supplementary Figure 4.** **Ricolinostat effect on T cells.**

A-D) Cytotoxic effect of 5 µM and 10 µM of ricolinostat on resting CD4+ (A, n=3), resting CD8+ (B, n=3), activated CD4+ (C, n=3) and activated CD8+ (D, n=3) T cells after 24, 48 and 72 hours of exposure. The bar diagrams show the percentage of viable (7-AAD neg) T cells determined by flow cytometry.

E-H) CD38 expression on resting CD4+ (E, n=3), resting CD8+ (F, n=3), activated CD4+ (G, n=3) and activated CD8+ (H, n=3) T cells before and after treatment with ricolinostat at 5 µM and 10 µM for 24, 48 and 72 hours.

A-H) CD4+ and CD8+ cells were isolated from n=3 healthy donors using magnetic bead isolation. Data are presented as mean values ± SD.

**Supplementary Figure 5. Ricolinostat effect on Jeko-1, Raji and Jurkat cell lines.**

A-C) Cytotoxic effect of ricolinostat on Jeko-1 cells (mantle cell lymphoma) (A, n=3 experiments), Raji cells (burkitt lymphoma) (B, n=3 experiments) and Jurkat cells (acute T-cell leukemia) (C, n=4 experiments) prior to and after ricolinostat treatment at 5 µM and 10 µM, respectively, for 24, 48 and 72 hours. The bar diagram shows the percentage of viable (7-AAD neg) cells determined by flow cytometry.

D-F) CD38 expression on Jeko-1 cells (D, n=3 experiments), Raji cells (E, n=3 experiments) and Jurkat cells (F, n=4 experiments) with and without ricolinostat treatment. The bar diagram shows CD38 expression before and after treatment with ricolinostat at 5 µM and 10 µM for 24, 48 and 72 hours.

A-F) Data are presented as mean values ± SD.

**Supplementary Figure 6. Ricolinostat effect on Daudi cell line and CDC control experiment.**

A) Viability of Daudi cells (n=3 experiments) prior to and after ricolinostat treatment at 5 µM and 10 µM, respectively, for 24, 48 and 72 hours.

B) CD38 expression on Daudi cells (n=3 experiments) with and without ricolinostat treatment. The bar diagram shows CD38 expression as normalized MFI before and after treatment with ricolinostat at 5 µM and 10 µM for 24, 48 and 72 hours.

C) The bar diagram shows the basal expression level of CD55 and CD59 on MM.1S cells compared to Daudi cells (n=3 experiments).

D) CD55 and CD59 expression as normalized MFI on Daudi cells (n=3 experiments) prior to and after ricolinostat treatment. The bar diagram shows CD55 and CD59 expression before and after treatment with ricolinostat at 5 µM for 24, 48 and 72 hours.

E) CDC using daratumumab against Daudi cells (n=3 experiments). Solvent control, IgG1 isotype control antibody (10 µg/ml) or daratumumab (10 µg/ml) and medium supplemented with fresh human serum (HS active) or heat-inactivated human serum (HS inactive) were added to induce CDC. The percentage of live Daudi cells was determined after 16 hours by flow cytometry. The bar diagram shows the percentage of viable (7-AAD neg) CD19+ Daudi cells.

A-E) Data are presented as mean values ± SD. P-values between indicated groups were calculated using Student´s t-test. ***p<.001, ****p<.0001.

**Supplementary Figure 7. ADCC experiments performed with the control antibody.**

A, B) ADCC against MM.1S (A, n=8 experiments) and OPM-2 (B, n=4 experiments) cells with and without ricolinostat treatment. Ricolinostat pre-treatment was performed for 48 hours at 5 µM. PBMC from healthy donors (effector-to-target ratio of 25:1) and control antibody were added at the indicated concentrations. Target cells stably express firefly luciferase and viability was analyzed after addition of luciferin substrate by bioluminescence after 24 hours.

Data are presented as mean values ± SD.

**Supplementary Figure 8. Daratumumab-mediated CDC and CD55/CD59 expression levels on myeloma cells after ricolinostat treatment.**

A-B) CDC against MM.1S (A, n=6 experiments) and OPM-2 (B, n=6 experiments) MM cells with and without ricolinostat treatment. Ricolinostat pre-treatment was performed for 48 hours at 5 µM, then daratumumab (0.1µg/ml) or control antibody (1µg/ml) and medium supplemented with fresh human serum (HS) were added to induce CDC. MM1.S and OPM-2 stably express firefly luciferase and viability was analyzed after addition of luciferin by bioluminescence after 16 hours.

C-E) CD55 and CD59 expression on MM1.S (C, n=4 experiments), OPM-2 (D, n=4 experiments) and primary MM (E, n=4 patients) cells prior to and after ricolinostat treatment. The bar diagrams show CD55 and CD59 expression before and after treatment with ricolinostat at 5 µM for 24 and 48 hours.

A-E) Data are presented as mean values ± SD. P-values between indicated groups were calculated using a Student´s t-test. **p<.005, ***p<.001, ****p<.0001

**Supplementary Figure 9. Ricolinostat effect on regulatory T cells.**

A) Cytotoxic effect of 5 µM (n=1 experiment) and 10 µM (n=3 experiments) of ricolinostat on regulatory T cells after 24 and 48 hours of exposure. The bar diagrams show the percentage of viable (7-AAD neg) regulatory T cells determined by flow cytometry.

B) CD38 expression on regulatory T cells before and after treatment with ricolinostat at 5 µM (n=1 experiment) and 10 µM (n=3 experiments) for 24 and 48 hours.

A-B) Regulatory T cells were isolated from n=3 healthy donors using two-step magnetic bead isolation. Data are presented as mean values ± SD.

**Supplementary Figure 10. *In vitro* ricolinostat treatment leads to enhanced CD38-expression on myeloma cells from a patient secondary refractory to daratumumab.**

The overlay histogram shows flow cytometric analysis of CD38-expression on primary MM cells cultured in the absence or presence of 5 µM of ricolinostat for 24 and 48 hours. Incubation time is indicated on the left, CD38 expression is indicated by MFI on the right.

**Supplementary Figure 11. Cytotoxic anti-myeloma effect of HDAC6 inhibitors.**

A-C) Viability of MM.1S (A, n=6 experiments), OPM2 (B, n=6 experiments) and U266 (C, n=3 experiments) cells before and after treatment with ricolinostat, ACY-241 and WT-161 at 1 µM, 5 µM and 10 µM. Bar diagrams show the percentage of viable (7-AAD neg) MM cells (CD38+/CD138+) determined by flow cytometry.

Data are presented as mean values ± SD. P-values between indicated groups were calculated using Student´s t-test. n.s = not significant, *p<.05, **p<.005, ***p<.001, ****p<.0001.
