# Supplementary material for: Upregulation of CD38 expression on multiple myeloma cells by novel HDAC6 inhibitors is a class effect and augments the efficacy of daratumumab
Source: Leukemia. 2020 Apr 29;35(1):201–14. doi: 10.1038/s41375-020-0840-y (PMC8318885; doi:10.1038/s41375-020-0840-y)
Supplement: Supplementary file 2 — Supplemental Table 1 [file 41375_2020_840_MOESM2_ESM.pdf]

## Supplemental Table 1: Patient characteristics

| patient identifier | age [years] | sex | type | cytogenetic aberrations | time from Dx [months] | clinical state | previous therapy |
|---|---|---|---|---|---|---|---|
| R01[a] | 58 | f | IgDλ | +1q21 | 70 | relapsed | 1: 4x RAD + CE + 2x HDMel/autoSCT<br>2: 2x VD<br>3: 3x VRD<br>4: 2x EloPomD<br>5: 13x Kd |
| R02[a,b] | 54 | m | κLC | ND | 77 | refractory | 1: 4x PAD + C + HDMel/autoSCT<br>2: 4x TCED<br>3: 12x Rd<br>4: 3x VRD<br>5: 2x DCEP<br>6: 2x Dara |
| R03 | 60 | m | IgGκ | none | 78 | refractory | 1: 2x VRD-PACE + IEV + 2x HDMel/autoSCT<br>2: 4x Pom-PAD<br>3: HDMel/autoSCT<br>4: 5x Pom-PAD<br>5: alloTx (MUD)<br>6: 4x R-VCD/DLI<br>7: 3x Pom-Kd |
| R04 | 58 | m | κLC | +1q21 | 19 | relapsed | 1: 3x VRD + CE + 2x HDMel/autoSCT |
| R05 | 74 | m | IgGλ | ND | NA | Dx | NA |
| R06[c] | 80 | m | IgGλ | del13q14, t(11;14), +1q21 | 24 | refractory | 1: 9x Dara-VMP + 9x Dara mono |
| R07 | 58 | f | IgGκ | del13q14, t(11;14), +1q21 | NA | Dx | NA |
| R08[a] | 67 | m | IgGκ | ND | 125 | refractory | 1: 3x VAD + CAD + 1x HDMel/autoSCT + ThalMT<br>2: 17 x KRd<br>3: 2x Pano-VD<br>4: 3x Ixa-Rd<br>5: 2x Venetoclax |
| R09 | 64 | m | κLC | ND | NA | Dx | NA |
| R10 | 54 | m | IgAλ | t(14;16), del13q14, del1p32, +1q21 | 12 | relapsed | 1: 3x VCD<br>2: 2x KRd<br>3: 1x DCEP + 2x HDMel/autoSCT |
| R11 | 81 | m | IgGλ | t(4;14), del13q14 | NA | Dx | NA |
| R12 | 76 | m | IgGκ | ND | 133 | refractory | 1: IEV + 2x HDMel/autoSCT<br>2: AUY-922/V (phase I/II)<br>3: 3x RAD<br>4: 3x Benda-RD<br>5: 9x VRD<br>6: HDMel/autoSCT<br>7: 6x Pom-VD<br>8: EloPomD |

[a] in these patients, *d*STORM analysis was performed

[b] data shown in Fig. 5

[c] data shown in supplementary Fig. 8

Abbreviations: Dx, primary diagnosis; ND, not done; RAD, lenalidomide/doxorubicin/dexamethasone; CE, cyclophosphamide/etoposide; HDMel/autoSCT, high dose melphalan and autologous stem cell transplant; VD, bortezomib/dexamethasone; VRD, bortezomib/lenalidomide/dexamethasone; EloPomD, elotuzumab/pomalidomide/dexamethasone; KD, carfilzomib/dexamethasone; PAD, bortezomib/doxorubicin/dexamethasone; C, cyclophosphamide; TCED, thalidomide/cyclophosphamide/etoposide/dexamethasone; Rd, lenalidomide/dexamethasone; DCEP, dexamethasone/cyclophosphamide/etoposide/cisplatin; Dara, daratumumab; PACE, cisplatin/doxorubicin/cyclphosphamide/etoposide; IEV, ifosfamide/epirubicin/etoposide; Pom, pomalidomide; alloTx, allogeneic stem cell transplantation; MUD, matched unrelated donor; R, lenalidomide; DLI, donor lymphocyte infusion; NA, not applicable; VMP, bortezomib/melphalane/prednisone; VAD, vincristine/doxorubicin/dexamethasone; CAD, cyclophosphamide/doxorubicin/dexamethasone; ThalMT, thalidomide maintenance; KRd, carfilzomib/lenalidomide/dexamethasone; Pano, panobinostat; Ixa, ixazomib; VCD, bortezomib/cyclophosphamide/dexamethasone; Benda, bendamustine.
