# Supplementary figures and images for: Upregulation of CD38 expression on multiple myeloma cells by novel HDAC6 inhibitors is a class effect and augments the efficacy of daratumumab
Source: Leukemia. 2020 Apr 29;35(1):201–14. doi: 10.1038/s41375-020-0840-y (PMC8318885; doi:10.1038/s41375-020-0840-y)

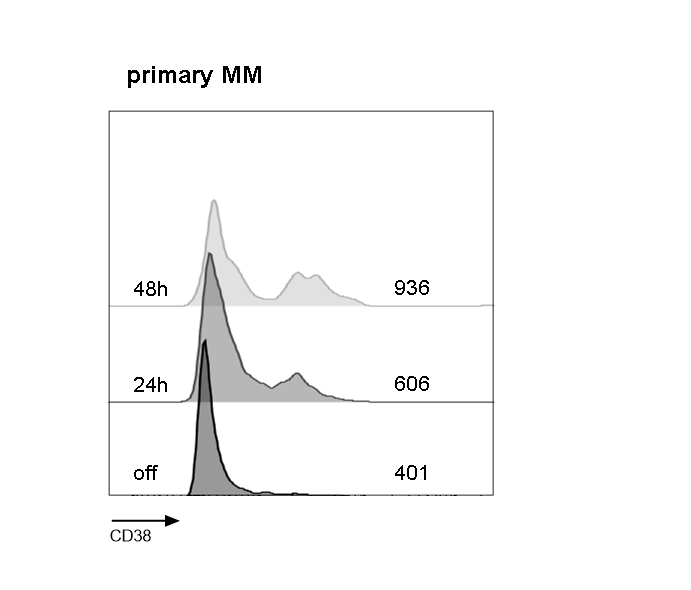

Supplement: Supplementary file 12 — Supplemental Figure 10 [file 41375_2020_840_MOESM12_ESM.tif]
